# Modulation of water diffusion by activation-induced neural cell swelling in *Aplysia Californica*

**DOI:** 10.1038/s41598-017-05586-5

**Published:** 2017-07-21

**Authors:** Yoshifumi Abe, Khieu Van Nguyen, Tomokazu Tsurugizawa, Luisa Ciobanu, Denis Le Bihan

**Affiliations:** 1 NeuroSpin, Bât 145, Joliot Institute, CEA-Paris-Saclay Center, Point Courrier 156, 91191 Gif-sur-Yvette, France; 2 University Paris-Saclay, 15 rue Georges Clemenceau, 91400 Orsay, France

## Abstract

Diffusion functional magnetic resonance imaging (DfMRI) has been proposed as a method for functional neuroimaging studies, as an alternative to blood oxygenation level dependent (BOLD)-fMRI. DfMRI is thought to more directly reflect neural activation, but its exact mechanism remains unclear. It has been hypothesized that the water apparent diffusion coefficient (ADC) decrease observed upon neural activation results from swelling of neurons or neuron parts. To elucidate the origin of the DfMRI response at cellular level we performed diffusion MR microscopy at 17.2 T in *Aplysia californica* buccal ganglia and compared the water ADCs at cellular and ganglia levels before and after neuronal activation induced by perfusion with a solution containing dopamine. Neural cell swelling, evidenced from optical microscopy imaging, resulted in an intracellular ADC increase and an ADC decrease at ganglia level. Furthermore, the intracellular ADC increase was found to have a significant positive correlation with the increase in cell size. Our results strongly support the hypothesis that the ADC decrease observed with DfMRI upon neuronal activation at tissue level reflects activation-induced neural cell swelling.

## Introduction

Diffusion functional magnetic resonance imaging (DfMRI)^1^ has been proposed as an alternative for blood oxygenation level dependent (BOLD)-fMRI^2^. BOLD-fMRI, which relies on the neurovascular coupling hypothesis and does not reflect neuronal activity directly^3, 4^, may fail in certain conditions which impair neurovascular coupling^5^. On the other hand, DfMRI is thought to be more directly linked to neuronal activation, as the diffusion MRI signal is exquisitely sensitive to minute changes occurring in the tissue microstructure upon various physiological or pathological changes^6^. Studies on rodents have evidenced that while the BOLD fMRI response is abolished by blocking the neurovascular response the DfMRI response is maintained, strongly suggesting that the DfMRI signal is not of vascular origin and that its mechanism differs from that of BOLD^7^. In addition, the DfMRI response is faster (time to reach the activation peak and time to return to baseline) than the hemodynamically driven BOLD signal response, as revealed by visual stimulation experiments in human subjects^1, 8^. While these results suggest that DfMRI is a more direct approach for functional imaging than BOLD-fMRI, the exact origin of the DfMRI response remains unclear.

Based on earlier reports that the water apparent diffusion coefficient (ADC) decreases in relation to cell swelling^1, 9–12^ and that neural swelling is one of the responses associated with neural activation^13–19^, it has been hypothesized that the decrease in the water ADC observed during neural evoked responses would originate from the dynamic swelling of neurons or neuron parts, in line with the so-called neuromechanical coupling hypothesis^6^. To investigate this hypothesis at cellular level, we performed diffusion magnetic resonance (MR) microscopy studies on the *Aplysia californica* buccal ganglia using an ultra-high magnetic field MRI (17.2 tesla). *Aplysia californica* is a key animal model in the neuroscience field^20^. The main advantage of using the buccal ganglia of *Aplysia californica* is the large size of its neurons which can be resolved and identified by MR microscopy^11, 21, 22^. Earlier reports showed that the water ADC decreased at the tissue level and increased inside neuron bodies when the cell was exposed to swelling inducers such as hypotonic solution or ouabain (an inhibiter of Na^+^/K^+^ pumps)^11^. In addition, the *Aplysia* neuronal preparation allows the investigation of neuronal activation in the absence of blood and neurovascular coupling effects. While the DfMRI signal acquired with a low degree of sensitization to diffusion (low b-value) in the mammalian brains contains a residual BOLD signal^23^, the absence of blood in the excised *Aplysia* ganglia guarantees the absence of any BOLD effect in the DfMRI signal acquired in this study. As electrophysiological^24^ and manganese enhanced MRI (MEMRI) studies^22^ have previously shown that dopamine promotes neural activation in the *Aplysia* buccal ganglia, we investigated water ADC changes induced by dopamine application using high resolution DfMRI. The associated neuronal swelling was demonstrated using optical microscopy.

## Results

### Dopamine stimulation induces cell swelling

**Figure 1:**
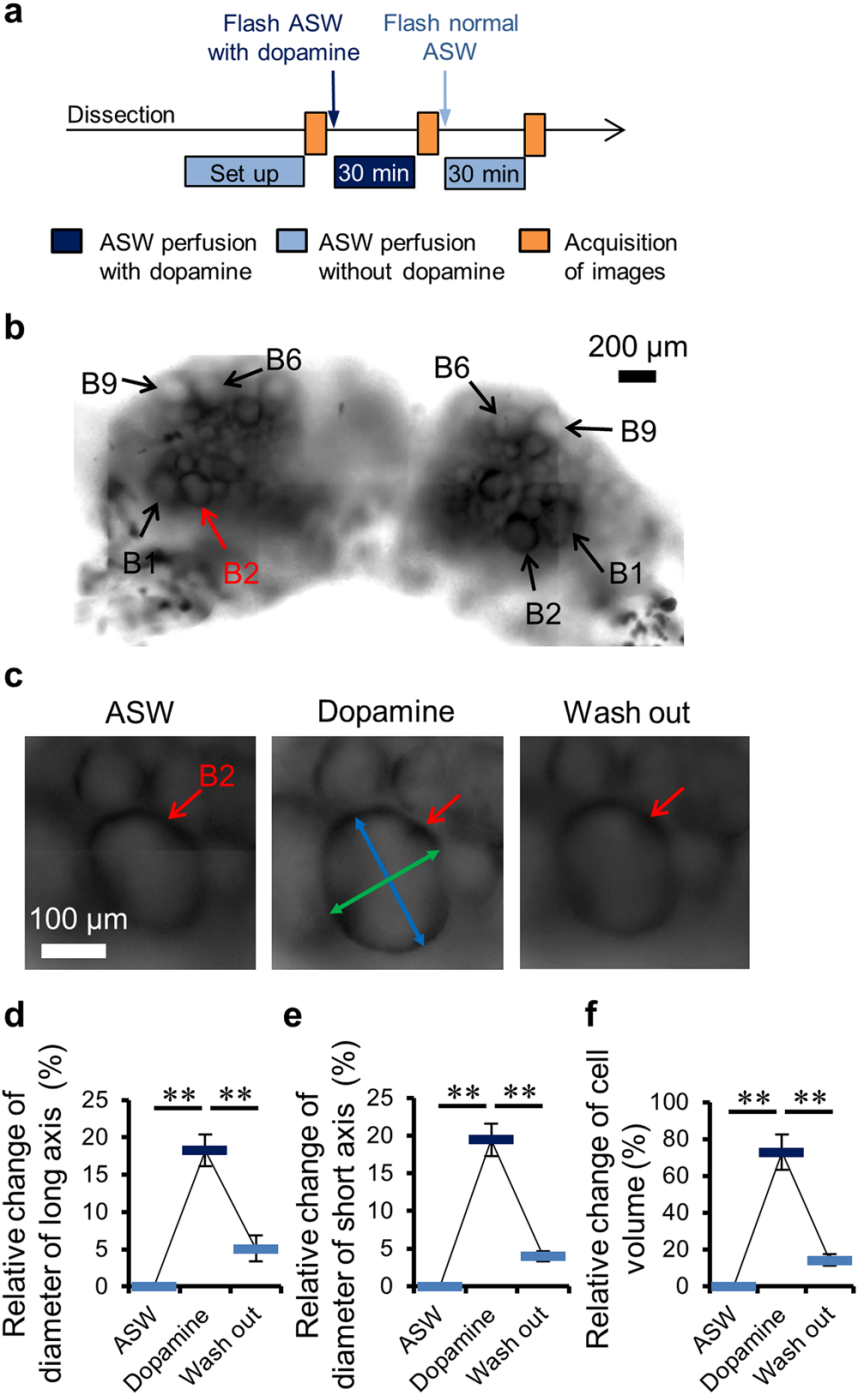
Dopamine stimulation induced neural cell swelling. (**a**) Time course of microscopy imaging. (**b**) Representative microscopy image of the entire buccal ganglia. (**c**) Representative microscopy images of somas of neuron B2 (red arrows) in ASW, after dopamine stimulation, and after washout. A blue arrow head shows the cell’s long axis and a green shows the short axis. The average relative change of the long axis (**d**), short axis (**e**), and volume (**f**) of neuron bodies for neurons B1, B2, B6, and B9 of each ganglion. These values were averaged over 26 neurons (7 B1 neurons, 7 B2 neurons, 6 B6 neurons, and 6 B9 neurons) in 4 *Aplysia* (each ganglion contains two neurons of each type). The images of cells with ambiguous contours were discarded. **p < 0.01 (Tukey-Kramer’s test). Bars plots exhibited mean ± SEM.

At first, cell swelling was observed by optical microscopy. Representative images of neuron B2 of the buccal ganglia showing cell swelling induced by dopamine stimulation are shown in Fig. 1b,c. Both the average cell diameters (long and short axes) and cell volume of neurons B1, B2, B6, and B9 were found to significantly increase after dopamine stimulation and to decrease after washing with artificial sea water (ASW), while remaining slightly above baseline level (Fig. 1d–f). The same behavior was observed for other neurons.

### Water ADC increases inside neurons and decreases at ganglia level upon cell swelling induced by dopamine stimulation

**Figure 2:**
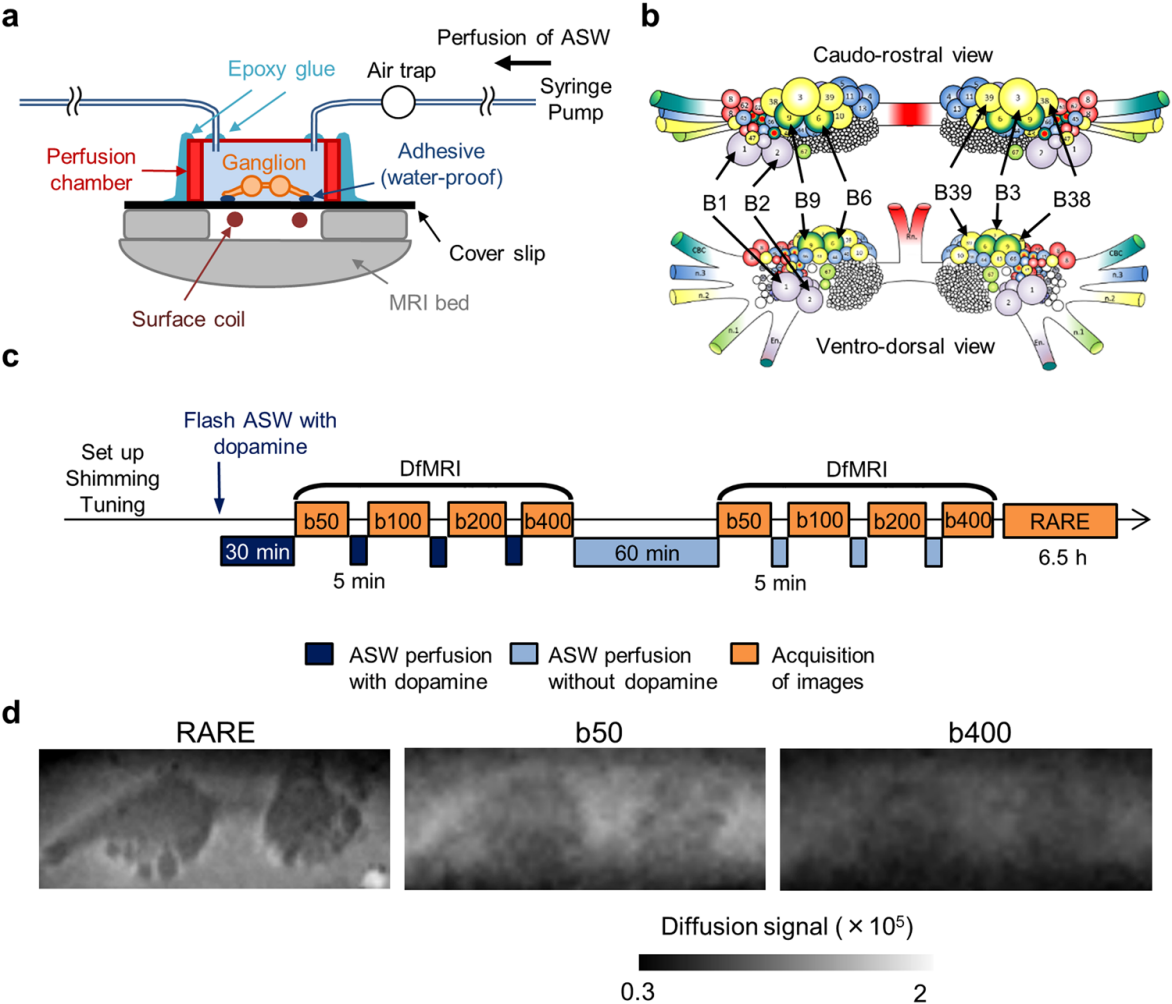
Experimental time course and perfusion system for MR microscopy. (**a**) Schematic diagram of the perfusion system used during MR microscopy experiments. (**b**) Schematic diagram of the buccal ganglia showing neurons B1, B2, B3, B6, B9, B38, and B39. (**c**) Time course of DfMRI experiments. 5 min-perfusion with dopamine +ASW or normal ASW was performed between diffusion acquisitions of each b-value (50, 100, 200, and 400 s/mm^2^). The 60 min-perfusion period after the dopamine stimulation and before the next acquisitions accounted for the time necessary for the ASW to reach the ganglia (approximately 30 min). (**d**) Representative images of RARE and DfMRI obtained with b-values = 50 and 400 s/mm^2^.

**Figure 3:**
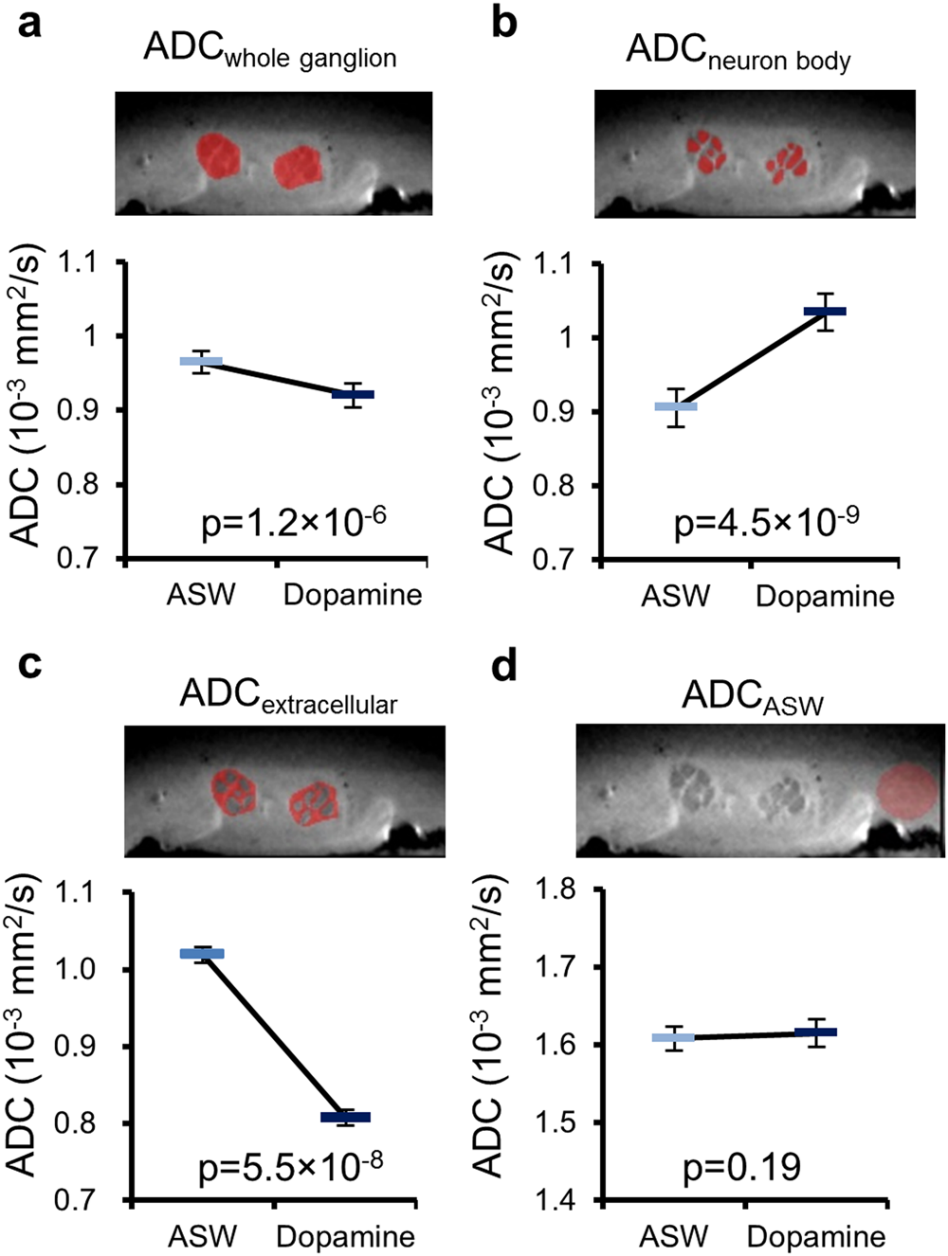
ADC decreased at ganglia level and increased at cellular level after dopamine stimulation. Average ADC changes after dopamine stimulation in the ganglia tissue (**a**), neuron bodies (**b**), extracellular region (**c**), and ASW regions (**d**). ROIs (red) of the ganglia tissue, neuron bodies, extracellular region, and ASW region were shown on the structural RARE image. The ADC values in the ganglia tissue were averaged over both sides of the ganglia in 7 *Aplysia* (14 ROIs). The ADC values of neuron bodies were averaged over all neurons (B1, B2, B3, B6, B9, B38, and B39) of each side of the 14 ganglia. The ADC value of ASW region was averaged over ASW ROIs in 7 samples. The statistical analysis was performed using a bilateral paired t-test. Bars plots exhibited mean ± SEM.

**Figure 4:**
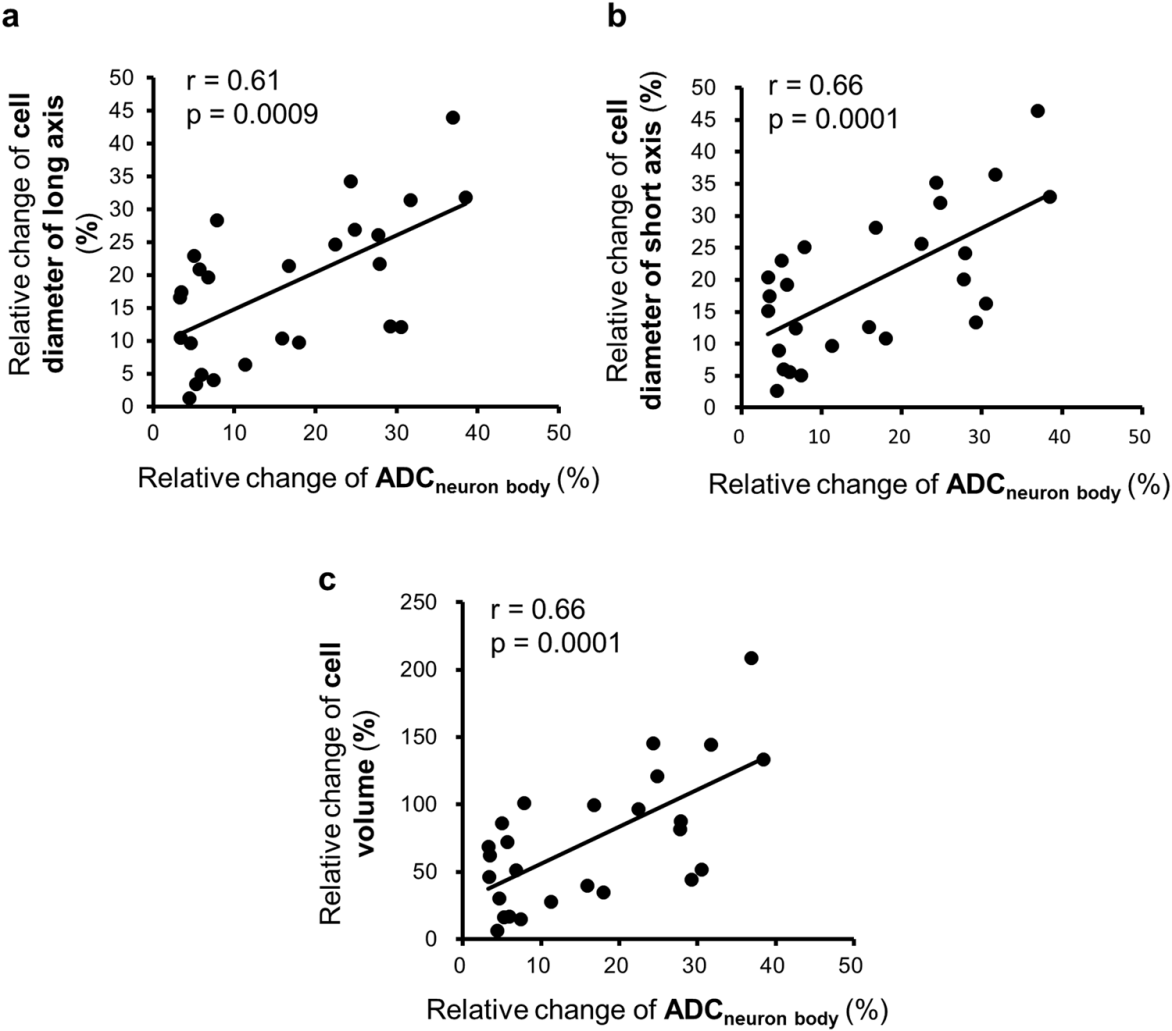
The cellular ADC increase was correlated with the cell size change. Scatter plots showing a correlation between the relative ADC change and the relative change of cell diameter of long axis (**a**), short axis (**b**) and cell volume (**c**) in neuron bodies of B1, B2, B6, and B9. 26 neurons of B1, B2, B6, and B9 in 4 *Aplysia*. Straight lines are intended to show the correlations between intracellular ADC and axes, volume.

The perfusion system we developed (Fig. 2a) allowed for ADC measurements under different perfusion conditions without removing the sample from the magnet bore. To minimize flow artifacts the diffusion weighted images were acquired under a stopped-flow condition (Fig. 2c). Each diffusion-weighted MRI acquisition was followed by a 10 min-perfusion period. The bilateral buccal ganglia regions and neuron bodies of B1, B2, B3, B6, B9, B38, and B39 (Fig. 2b) were identified on the structural relaxation enhancement (RARE) images. The ADC was calculated before and during dopamine perfusion, as well as after washout. Representative images of RARE and DfMRI are shown in Fig. 2d. The average ADC over the ganglia and the extracellular regions was significantly decreased by dopamine stimulation (Fig. 3a,c); from 0.96 ± 0.01 × 10^−3^ mm^2^/s (ASW) to 0.92 ± 0.02 (dopamine) and from 1.02 ± 0.01 (ASW) to 0.81 ± 0.01 (dopamine), respectively. On the other hand, the ADC averaged over neuron bodies was significantly increased by dopamine stimulation (0.91 ± 0.03 for ASW and 1.03 ± 0.03 for dopamine, Fig. 3b). The values of the ADCs found in each neuron body are provided in Table 1. As a control, no change in ADC was observed in regions containing only ASW (Fig. 3d). The dopamine-induced cellular ADC increase in neurons B1, B2, B6, and B9 was found to be significantly correlated with the increase in the cell diameters (long and short axes) and cell volume of those neurons (Fig. 4).

**Table 1:** ADC values inside neurons, cell diameters, and volume with and without dopamine stimulation.

Cell	Cell diameter of long axis (μm)		p value (paitd t-test)	Cell diameter of short axis (μm)		p value (paitd t-test)	Cell volume (×10^7^ μm^3^)		p value (paitd t-test)	ADC (10^−3^ mm^2^/s)		p value (paitd t-test)
	ASW	Dopamine		ASW	Dopamine		ASW	Dopamine		ASW	Dopamine	
B1	194 (11)	240 (12)	0.0003	171 (9)	209 (12)	0.0006	2.51 (0.40)	4.62 (0.77)	0.0005	0.91 (0.03)	1.06 (0.03)	0.0046
B2	169 (14)	204 (13)	0.0024	145 (14)	180 (15)	0.0017	1.69 (0.35)	3.02 (0.53)	0.0009	0.89 (0.05)	1.08 (0.07)	0.0021
B3	—	—	—	—	—	—	—	—	—	0.91 (0.03)	1.07 (0.03)	0.0008
B6	187 (5)	211 (3)	0.0062	169 (5)	192 (5)	0.0046	2.24 (0.19)	3.28 (0.19)	0.0044	0.92 (0.03)	1.07 (0.03)	0.0008
B9	171 (8)	189 (8)	0.0052	150 (8)	169 (9)	0.0025	1.69 (0.26)	2.34 (0.34)	0.0039	0.96 (0.05)	1.15 (0.06)	0.0022
B38	—	—	—	—	—	—	—	—	—	0.95 (0.04)	1.13 (0.03)	0.0026
B39	—	—	—	—	—	—	—	—	—	0.82 (0.08)	0.94 (0.09)	0.0022

The values were averaged over neurons on both sides of the ganglia in 4 *Aplysia* for microscopy data and 7 *Aplysia* for the ADC. The values are showed as Mean (±SEM). It was not possible to measure neurons B3, B38, and B39 with optical microscopy as they were located behind neurons B6 and B9.

## Discussion

In this study, we have developed a perfusion system for performing MR microscopy studies using a 17.2 T MRI scanner for the following three purposes: 1) to keep the *Aplysia* buccal ganglia in good physiological condition; 2) to fix the ganglia in the same position with respect to the radio frequency (RF) coil during perfusion; 3) to compare the DfMRI signals (water ADC) of the buccal ganglion neurons before and after perfusion with the dopamine neurotransmitter without removing the sample from the magnet bore. Our results show that dopamine stimulation induces an intracellular ADC increase and an ADC decrease in the whole ganglion. These ADC changes were significantly correlated with neuronal volume changes induced by dopamine activation. Our results are consistent with a previous report showing that the water ADC decreased at ganglia tissue level and increased inside neuron bodies after changes in neuronal bodies induced by extra-physiological exposure to hypotonic shock and ouabain^11^.

Based on previous MEMRI and electrophysiological studies, dopamine induces neural activation of the *Aplysia* buccal ganglia^22, 24^. In our current study, optical microscopy measurements demonstrated that the neurons’ cell body diameters volumes were increased by dopamine stimulation, in agreement with earlier reports demonstrating that neural swelling is associated with neuronal activation^11, 13–18, 25–27^. Moreover, as a confirmation of cell viability, we observed that the volume of swollen neuronal bodies decreased back after washing out the dopamine solution.

While the intracellular ADC increase likely reflects a dilution effect in cytoplasm upon cell swelling^11^, the mechanism of the ADC decrease at ganglia or tissue level upon cell swelling remains putative at this stage. Possible explanations include an increased tortuosity within the reduced extracellular space^28–30^ or an increase of the neuron membrane surface leading to a volumetric increase in the water molecular layer bound electrostatically to the membranes and adjacent macromolecules^11, 14, 31^. In our MR microscopy study, the ADC change in subcellular compartments, such as nucleus, cytoplasm, and cellular membrane, could not be assessed due to low signal-to-noise ratio (SNR) and limited spatial resolution. A previous study revealed, however, that ADCs and volumes of nucleus and cytoplasm were increased under hypotonic stress and ouabain exposure^11^. Much higher spatial resolution studies will be necessary to elucidate whether water diffusion near cell membranes is lower than within the cytoplasm.

Although those results may not be directly extrapolated to the mammalian brain, the *Aplysia* neural tissue shares the feature observed in the mammalian brain, namely that neuronal activation is accompanied by cell swelling, however without the confounding effects of blood flow. Our results clearly show that this activation-driven cell swelling leads to a decrease of water diffusion at the neural tissue level, as we have hypothesized earlier^1, 9, 10, 12, 32, 33^, although water diffusion increases inside swelling neurons, in line with the neuromechanical coupling hypothesis^6^.

## Conclusion

Upon stimulation with dopamine of *Aplysia californica* neuronal tissue diffusion MRI microscopy revealed a water diffusion (ADC) increase inside neuron bodies, which correlated positively with cell swelling, and a decrease at ganglia tissue level, in agreement with early findings on the mammalian brain. Our study confirms the hypothesis that the water ADC decrease observed in neural tissue is strongly correlated to activation-induced cell swelling.

## Methods

### Tissue preparation of *Aplysia* buccal ganglia

Seven juvenile *Aplysia californica* (National Resource for *Aplysia*; Miami, FL, USA) were used in total. On three samples we performed only MR microscopy while on the other four we also acquired optical images. *Aplysia* were anesthetized by injection of an isotonic magnesium chloride solution (360 mM MgCl_2_ and 10 mM HEPES, pH = 7.5). The animals were dissected on a sylgard-coated petri dish (Dow Corning; Midland, MI, USA) filled with cold ASW (450 mM NaCl, 10 mM KCl, 30 mM MgCl_2_, 20 mM MgSO_4_, 10 mM CaCl_2_, 10 mM HEPES, pH = 7.5, osmolarity = 1090 mOsm/l) to isolate the buccal ganglia. All chemicals were purchased from Sigma-Aldrich (St. Louis, MO, USA).

### Optical microscopy imaging

Optical microscopy imaging was performed at room temperature before the MR microscopy study. The isolated buccal ganglia were placed inside a glass chamber (Thermo Fisher Scientific; Rochester, NY, USA) provided with two silicon perfusion tubes attached at each side. The ganglia were fixed inside the chamber using hand-made stainless steel anchors made out of 0.1 mm thick threads placed at 1 mm spacing. The ASW solution was supplied from one side of the chamber at a flow rate of 2.5 ml/h using a syringe pump (Harvard Apparatus; Holliston, MA, USA) and aspirated from the other side at approximately identical flow rate using a peristaltic pump (Watson-Marlow; Wilmington, MA, USA) to keep the same depth of ASW solution. The ASW solution containing dopamine (pH = 7.5, osmolarity = 1091 mOsm/l) was prepared by adding 0.05 mM dopamine hydrochloride (Sigma-Aldrich; St. Louis, MO, USA) and 0.05 mM ascorbic (antioxidant) acid (Sigma-Aldrich; St. Louis, MO, USA). The time course of optical microscopy experiments is described in Fig. 1a. Before starting the image acquisition, the isolated buccal ganglia were perfused with fresh ASW for 30 min. Three sets of microscopy images, 15 μm slice thickness, were acquired: 1) after perfusion with ASW; 2) after 30 min-perfusion with dopamine-ASW solution; and 3) after 30 min-perfusion (washout) with ASW. The acquisitions were performed using an Axio Observer Z1 microscope (Zeiss; Marly-le-Roi, France) with a 10X objective and Zeiss Axio Vision software. The 30 min-perfusion time was chosen in order to allow dopamine to penetrate the outer connective tissue sheath of the ganglia and reach the neurons^24^.

Measurements of cells’ long and short axes (Fig. 1c) were performed using ImageJ software (NIH; Bethesda, MD, USA). These long and short axes were defined as the longest and shortest axes which were orthogonal, respectively (Fig. 1c). Four big neurons (B1, B2, B6, and B9) of the buccal ganglia were identified on each microscopy image. Other neurons (B3, B38, and B39) were not accessible with optical microscopy as they are located behind B6 and B9 (Fig. 2b). The cell volume (V) was estimated with the following formulas:

1 $$V=\frac{4}{3}\pi b{a}^{2}$$

where a is the short axis and b is the long axis. The significance of the cell diameters and cell volume changes induced by dopamine stimulation was quantified by performing a Tukey-Kramer’s test. The relative changes in cell axes and cell volume of neurons B1, B2, B6, and B9 were calculated as (X_dopamine_ − X_ASW_)/X_ASW_ × 100, where X_dopamine_ and X_ASW_ are the cell axes or volume after dopamine stimulation after and ASW washout, respectively.

### Perfusion system and MR microscopy

The buccal ganglia which were used in the optical microscopy study were transferred from the glass chamber used for optical microscopy imaging to the perfusion system for MR microscopy (Fig. 2a). The bilateral nerves of the buccal ganglia were fixed on the cover slip (22 × 50 mm, No. 1.5; Scientific Laboratory Supplies Ltd.; Hessle, UK) using a water-proof, biological silicone elastomer adhesive (World Precision Instruments, Inc.; Sarasota, FL, USA) to prevent movement during perfusion. The perfusion chamber (2.5 mm depth, 150 μl chamber volume; Sigma-Aldrich) was provided with two small holes (1.5 mm diameter) in which the perfusion silicon tubes were inserted (length 3 m). Air traps were attached to the tubes between the perfusion chamber and the syringe pump to remove air bubbles. The ASW and dopamine solutions were supplied at a flow rate of 2.5 ml/h using a syringe pump (Harvard Apparatus; Holliston, MA, USA). The MR acquisitions were performed under stopped flow condition.

MR microscopy was performed at 19 °C on a 17.2 T MRI scanner (Bruker BioSpin; Ettlingen, Germany) equipped with 1 T/m gradients. The radio frequency (RF) transceiver was a home-built surface micro-coil consisting of a single loop (inner diameter of 4.2 mm). The time course of MR microscopy experiments is described in Fig. 2c. DfMRI images were acquired with the following parameters: 2D-spin echo diffusion weighted sequence; TR = 2000 ms, TE = 13.11 ms, FOV = 0.4 × 0.16 cm^2^, Matrix = 80 × 32, Number of slices = 8, Slice thickness = 0.1 mm Resolution = 50 × 50 × 100 μm^3^, diffusion time δ = 2 ms, ⊿ = 7 ms, 4 b-values = 50, 100, 200, 400 s/mm^2^, Diffusion gradient encoding direction = (X = 1, Y = 1, Z = 1), Number of averages = 8, Scan time per b-value = 10 min. A first set of DfMRI images was acquired after 30-min perfusion with dopamine solution. The acquisition lasted for 40 min which coincides with the duration of dopamine-induced neural response^24^. A second set of DfMRI images was acquired after washout with ASW for 60 min, knowing that the ASW reached the ganglia in approximately 30 min after starting the perfusion. Although ganglia oxygenation was ensured through continuous perfusion, as an extra precaution the DfMRI images under dopamine  + ASW were obtained before those under pure ASW to avoid potentially confounding factors related to hypoxia induced ischemia. Finally, T2 weighted structural images were also acquired with the following parameters: 3D-relaxation enhancement (RARE) sequence; TR = 2000 ms, TE = 16 ms, RARE factor = 4, FOV = 0.4 × 0.16 × 0.3 cm^3^, Matrix = 160 × 64 × 60, Resolution = 25 × 25 × 50 μm^3^, Number of averages = 12, Scan time = 6 h 24 min. The total duration of the experiments from the dissection of the *Aplysia* until the final MR image was approximately 12.5 h. This duration is well within the ganglion expected survival time of 48 h^22^.

### ADC analysis

All processing was performed using Matlab (The MathWorks; MA, USA). For each sample, the structural RARE image and the DfMRI images were co-registered to the same position. For segmentation of cellular and ganglia region, the structural RARE images were displayed as successive slices in Matlab. The neuron bodies (B1, B2, B3, B6, B9, B38, B39) and ganglia region of each side were manually segmented on each slice, based on signal contrast of the RARE image. The positions of these large neurons were easy to identify in the optical and MR microscopy images^11, 21, 22^. The MR signals for each b-value were extracted from the DfMRI images and averaged within each ROI for ADC estimation. The ADCs were calculated by fitting S/So(b) = exp(-bADC), where S/So(b) is the signal attenuation at each b value. The ADCs were estimated individually for each neuron body and each bilateral ganglion. The ADC was also calculated in an ROI placed in the ASW region. The significance of the ADC changes induced by dopamine stimulation was quantified using a bilateral paired Student’s t-test, performed on the series of ADC values before and after dopamine stimulation. For neurons B1, B2, B6, and B9, the relative change in ADC was calculated as (ADC_dopamine_ − ADC_ASW_)/ADC_ASW_ × 100, where ADC_dopamine_ and ADC_ASW_ are the ADC values obtained after dopamine stimulation and after washout, respectively. The relative change in ADC, cell axes, and volume were obtained from 4 neurons (B1, B2, B6, and B9) in the ganglia examined by both optical microscopy and MRI. Then, the correlation between the relative increase in ADC and the relative change in cell axes and volume were quantified using the Pearson product-moment correlation coefficient. To provide some indication of the signal level in our DfMRI image data without dopamine solution, signal-noise ratios (SNRs) were estimated by dividing the average signal in whole ganglion region by the standard deviation in the noise region. Averaged SNR values (mean ± sem) were 11.17 ± 0.92 for b50, 10.86 ± 1.20 for b100, 8.98 ± 0.72 for b200 and 7.05 ± 0.84 for b400 (n = 7).
